# The Role of the Family Microenvironment in Adolescents’ Physical Activity and Nutritional Status

**DOI:** 10.3390/children13101315

**Published:** 2026-09-27

**Authors:** Ilona Karácsony, Gabriella Hideg-Fehér

**Affiliations:** 1 Institute of Basics of Health Sciences, Midwifery and Health Visiting, Faculty of Health Sciences, University of Pécs, 9700 Szombathely, Hungary; ilona.karacsony@etk.pte.hu; 2 Faculty of Health and Sport Sciences, Széchenyi István University, 9026 Győr, Hungary; 3 Institute of Physiotherapy and Sport Science, Faculty of Health Sciences, University of Pécs, 7622 Pécs, Hungary

**Keywords:** family microenvironment, perceived social support, physical activity, nutritional status, weight perception, overweight and obesity

## Abstract

**Highlights:**

**What are the main findings?**

- Only 21.7% of adolescents reported at least 60 min of moderate-to-vigorous physical activity on all seven days of the previous week, while 26.7% had overweight or obesity.
- Nearly half of the adolescents inaccurately perceived their weight status; exploratory coefficients suggested possible associations between selected family characteristics and BMI-derived weight status, while higher perceived peer support was associated with lower odds of weight underestimation.

**What are the implications of the main findings?**

- Future adolescent health-promotion research may examine integrated approaches addressing physical activity, nutrition education, and accurate weight perception.
- Future school- and family-based interventions may consider involving parents, teachers, and peers, with additional attention to adolescents from potentially disadvantaged family environments.

**Abstract:**

**Background**: Adolescents’ physical activity, BMI-derived weight status, and weight perception are associated with their current and future health. This study examined the associations of family microenvironmental characteristics and perceived social support with these outcomes. **Methods**: A cross-sectional survey using convenience sampling was conducted among 411 Hungarian secondary school students aged 14–18 years. Physical activity was assessed using HBSC-based self-report items capturing weekly frequency and an operationalized indicator combining activity intensity and duration. Perceived social support was measured using the MSPSS. BMI-derived weight status was determined using age- and sex-specific percentiles calculated from self-reported height and weight. Chi-square tests and logistic regression models were applied. **Results**: Family background characteristics and perceived social support were not significantly associated with the examined physical activity indicators. Overall, 57.4% of participants had normal weight, while 26.7% were classified as having overweight or obesity. Only 55.2% accurately perceived their BMI-derived weight status. Exploratory coefficients suggested possible associations between selected family characteristics and BMI-derived weight status, although the corresponding overall models were not statistically significant. Higher perceived peer support was associated with lower odds of weight underestimation, whereas the association between perceived family support and weight overestimation did not reach statistical significance. **Conclusions**: Exploratory findings suggested possible associations of selected family and psychosocial characteristics with BMI-derived weight status and weight perception. Given the cross-sectional design, convenience sampling, self-reported anthropometric data, use of national BMI reference values, and limited explanatory performance of several models, the findings require cautious interpretation and should not be considered evidence of causal relationships.

## 1. Introduction

Adolescence is a formative period during which health-related attitudes and behaviors develop with potential long-term implications for adult health and quality of life. Physical activity, dietary behavior, and body-image-related attitudes established during this period are associated with the subsequent risk of chronic noncommunicable diseases [1]. Regular physical activity supports cardiovascular and metabolic health, bone development, mental health, and psychosocial well-being [2]; nevertheless, physical activity commonly declines during adolescence while sedentary behavior increases [3].

The current WHO guidelines recommend that children and adolescents engage in an average of at least 60 min per day of moderate-to-vigorous physical activity across the week [4]. HBSC surveys assess physical activity frequency by asking adolescents on how many days during the previous week they engaged in at least 60 min of moderate-to-vigorous physical activity. Across the 2010, 2014, and 2018 HBSC survey waves, fewer than 20% of adolescents reported this amount of activity on all seven days [5,6]. The 2022 Hungarian HBSC survey reported a similarly low proportion of 21.3% [7]. Although Hungarian adolescents may perform more favorably than their international peers on certain physical-activity indicators [8], regular leisure-time physical activity of sufficient intensity remains limited. In the present study, physical activity was therefore examined using two complementary indicators: weekly frequency and an operationalized indicator combining duration and intensity. The latter was based on previous research in which at least three hours per week of leisure-time physical activity at an intensity sufficient to induce sweating was classified as adequate [7,9]. This indicator does not represent the overall “quality” of physical activity; rather, it captures functional characteristics related to duration, intensity, and health-relevant exertion [10].

Physical inactivity should also be considered in relation to adolescent nutritional status. The prevalence of childhood and adolescent overweight and obesity has increased internationally [11], with important implications for later cardiometabolic health and chronic disease risk [12]. Weight status is shaped by multiple interacting biological, psychological, social, and environmental determinants [13], including genetic susceptibility, dietary habits, physical activity, sedentary behavior, screen use, and sleep [14,15]. Accordingly, adolescent physical activity and nutritional status are interrelated but distinct health-related outcomes that should be examined within their immediate social context.

The ecological approach provides a useful framework for organizing these contextual influences. Sallis et al. proposed that physical activity is shaped by biological and demographic characteristics, psychological and behavioral factors, and the sociocultural and physical environment [16]. Hungarian evidence similarly indicates that young people’s lifestyle-related attitudes should be interpreted in relation to their social and demographic circumstances [17]. Within this broader framework, the family represents the adolescent’s most immediate social environment, while peers, schools, place of residence, and wider social conditions provide additional context. Parental socioeconomic background may also have lasting associations with psychological adjustment and health-related behavior [6].

In the present study, the term “family microenvironment” refers specifically to the structural and psychosocial characteristics of the family as the adolescent’s immediate social environment. Its structural dimension comprises family structure, family size, the number and sex of siblings, and parental educational attainment, whereas its psychosocial dimension is represented by perceived family support. Parental educational attainment was treated as an indicator of family socioeconomic resources, while family structure and sibling characteristics represented aspects of relational structure. Place of residence was considered a sociodemographic characteristic of the broader social and physical context, and perceived peer support was examined as a complementary psychosocial factor outside the family. Thus, the ecological perspective was used to organize the variables examined rather than to test a comprehensive ecological model.

Families may influence adolescent health behavior through interconnected structural and psychosocial pathways. Family resources, role modeling, emotional support, communication, shared leisure activities, and opportunities for participation may jointly shape physical activity and weight-related behaviors [18]. Hungarian research has also indicated that parental influence on sport selection and specialization may vary according to the family’s financial investment in sport [19]. These findings support examining family characteristics as a related set of contextual factors rather than as isolated determinants.

Parental educational attainment is among the most frequently investigated structural characteristics of the family environment. Higher parental education has generally been associated with more favorable physical activity and sports-participation patterns, whereas lower educational attainment has sometimes been associated with less favorable weight status [20,21,22]. These associations may operate through health-related knowledge, material resources, parental modeling, and the opportunities available to adolescents. Evidence concerning family structure and sibling characteristics is less consistent. Some studies have reported lower physical activity among adolescents living in single-parent or reconstituted families, while others have found that associations with family structure vary according to age and sex [23,24]. Similarly, siblings may provide opportunities for shared activity, but findings concerning sibling number, sibling sex, household size, and physical activity remain heterogeneous [21,24,25,26].

The psychosocial dimension of the family microenvironment may be equally relevant. Perceived family support can include emotional security, acceptance, trust, communication, encouragement, and practical assistance. Although several studies have linked stronger family support with healthier lifestyle behaviors, the findings are not entirely consistent [27,28,29,30,31,32]. Family support should therefore be considered alongside structural family characteristics and the broader social environment rather than interpreted as an isolated influence. Weight perception represents another important aspect of adolescent health. Adolescents’ willingness to adopt weight-related lifestyle changes may depend partly on whether they perceive their own weight status accurately, making weight misperception a potential barrier to preventive efforts [33]. A substantial proportion of children and adolescents with overweight or obesity perceive their weight as normal [34,35]. In the United States, weight-status misperception has been reported among 77% of children with overweight and 43% of those with obesity [36], and substantial levels of inaccurate perception have also been observed in other adolescent populations [37]. The direction of misperception commonly differs by sex: boys are more likely to underestimate their weight, whereas girls are more likely to overestimate it [38,39].

Weight perception is associated not only with BMI-derived weight status but also with social norms, gender-related expectations, communication, and psychosocial support. Ben-Yaish et al. found associations between weight misperception, family support, parent–child communication, and peer support [40]. Supportive family relationships and emotional security have also been linked to adolescents’ health perceptions, self-esteem, and subjective well-being [41,42]. More broadly, parental communication, feedback, and shared health-related experiences may help shape adolescents’ body image and responses to social expectations [43,44]. However, relatively few studies have jointly examined structural family characteristics, family and peer support, BMI-derived weight status, and the accuracy of adolescents’ weight perception.

Previous research has therefore provided extensive evidence concerning individual family and psychosocial correlates of physical activity, weight status, and weight perception, but these outcomes and explanatory factors have often been studied separately. In particular, limited attention has been paid to the joint examination of structural and psychosocial characteristics of the family microenvironment in relation to both a frequency-based indicator of physical activity and an operationalized indicator combining activity duration and intensity, together with BMI-derived weight status and subjective weight perception.

The aim of the present study was to examine how the structural and psychosocial characteristics of the family microenvironment are associated with adolescents’ physical activity, BMI-derived weight status, and subjective weight perception. The structural characteristics examined were family structure, family size, the number and sex of siblings, and parental educational attainment. Perceived family support represented the psychosocial dimension of the family microenvironment, while place of residence and perceived peer support were treated as contextual factors outside the immediate family microenvironment.

Accordingly, the following research questions were formulated:

1. How are the structural characteristics of the family microenvironment associated with adolescents’ physical activity frequency and with an operationalized adequacy indicator combining activity duration and intensity?
2. Is perceived family support associated with adolescents’ physical activity frequency and with an operationalized adequacy indicator combining activity duration and intensity?
3. Are the structural and psychosocial characteristics of the family microenvironment associated with adolescents’ BMI-derived weight status and the accuracy of their subjective weight perception?

## 2. Materials and Methods

This quantitative, cross-sectional study was conducted in general secondary schools providing full-time education in the Western Transdanubian region of Hungary. A one-time convenience sampling procedure was used. Students enrolled in four-year, full-time general secondary education were eligible to participate, whereas students with private student status were excluded. The primary sampling units were classes selected using systematic random sampling, and all students in the selected classes were invited to participate. Data were collected during regular lessons using a group-administered procedure. Students independently completed anonymous paper-based questionnaires following standardized instructions. Before completing the questionnaires, each class received detailed information about the purpose and procedure of the study and was given an opportunity to ask questions. Some of the participating schools had also been involved in an earlier survey. Because both data collections were anonymous and no persistent participant identifiers were used, possible individual-level overlap between the samples could not be determined.

The questionnaire used in this study included both self-developed items and items adopted from standardized instruments. The self-developed questions assessed sociodemographic characteristics and the objective and subjective nutritional status of the participants. Maternal and paternal educational attainment were examined separately. The original response options were grouped into three analytical categories: (1) vocational or vocational secondary education, (2) upper secondary education culminating in a school-leaving examination, and (3) tertiary education. “I do not know” responses were treated as missing data. Height and body weight were self-reported and used to calculate body mass index (BMI = body weight [kg]/height^2^ [m^2^]). Because BMI varies by age and sex during childhood and adolescence, sex- and age-specific BMI percentiles based on the Hungarian reference values published by Ágfalvi et al. [45] were used to determine nutritional status. The Hungarian reference system distinguishes optimal weight status (10th–90th percentiles), lower and upper warning zones (3rd–<10th and >90th–97th percentiles), and clinically relevant undernutrition or obesity (<3rd and >97th percentiles, respectively) [45]. For the present analyses, these categories were collapsed into three BMI-derived weight-status groups: thinness (<10th percentile), normal weight (10th–90th percentiles), and overweight/obesity (>90th percentile). Thus, the lower warning zone and clinically relevant undernutrition were combined into the thinness category, while the upper warning zone and obesity category were combined into the overweight/obesity category. To improve international comparability, BMI-derived weight status was also categorized in a supplementary analysis using the 2007 WHO BMI-for-age reference. Participants were classified as having thinness (<−2 SD), normal weight (≥−2 SD to ≤+1 SD), or overweight/obesity (>+1 SD); the latter category combined overweight (>+1 SD to ≤+2 SD) and obesity (>+2 SD). Prevalence estimates obtained using the two reference standards are presented in Supplementary Table S3. A standardized data collection protocol was used for the self-reporting of anthropometric data to minimize potential measurement bias. Participants were asked to report the height and body weight values communicated to them following their most recent school health screening. These data were not obtained from direct measurements performed by healthcare professionals or from official school health records; therefore, they were treated as self-reported anthropometric data in the present study. This approach may reduce the measurement accuracy of the anthropometric data and, particularly among adolescents, does not eliminate the possibility of recall or self-report bias. Although self-reported anthropometric data among children and adolescents may involve a slight overestimation of height and underestimation of body weight, previous studies have demonstrated that such data have adequate reliability for use in population-level epidemiological research [46].

Subjective nutritional status was assessed using a five-point Likert-type item (“How do you perceive yourself?”), with the response options “much too thin,” “a little too thin,” “about the right size,” “a little too fat,” and “much too fat.” Subjective weight perception was compared with BMI-derived weight status based on the Hungarian age- and sex-specific BMI percentiles. For this comparison, the responses “much too thin” and “a little too thin” were classified as perceived thinness, “about the right size” as perceived normal weight, and “a little too fat” and “much too fat” as perceived overweight. Participants were then classified into three groups: accurate perception, underestimation, and overestimation. Accurate perception indicated agreement between the perceived and BMI-derived weight-status categories; underestimation indicated that the perceived category was lower than the BMI-derived category; and overestimation indicated that the perceived category was higher than the BMI-derived category. Perceived social support was measured using the validated Hungarian version of the Multidimensional Scale of Perceived Social Support (MSPSS) [47]. The questionnaire comprises 10 statements rated on a five-point Likert scale (1 = not at all; 5 = completely). The five-point response format and the 10-item structure were retained from the validated Hungarian version and were not introduced as study-specific modifications. This version was selected because it had been adapted and validated for use in a Hungarian population. Higher scores indicate greater perceived social support. The original questionnaire distinguishes three subscales (family, friends, and significant others); however, the present study analyzed the family and friends dimensions. Both subscales demonstrated adequate internal consistency (Cronbach’s alpha: family = 0.811; friends = 0.878). Physical activity was assessed using validated items from the international Health Behaviour in School-aged Children (HBSC) study [5,7]. Physical activity was assessed using two complementary indicators. Physical activity frequency was determined using the classification applied in the Health Behaviour in School-aged Children (HBSC) study [7]. Participants were categorized according to the number of days during the previous seven days on which they had engaged in at least 60 min of moderate-to-vigorous physical activity, including activity performed during school physical education classes. For the frequency-based indicator, participants were classified according to the number of days on which they reported at least 60 min of moderate-to-vigorous physical activity: all 7 days, 5–6 days, 3–4 days, or 0–2 days during the previous week. This HBSC-based frequency indicator was not interpreted as a direct measure of adherence to the current WHO recommendation, which is expressed as an average of at least 60 min per day across the week. The second indicator jointly reflected physical activity intensity and duration. Participants indicated how many hours per week they engaged in leisure-time physical activity at an intensity sufficient to induce sweating. The original six response categories, ranging from “none” to “7 h or more,” were transformed into a dichotomous variable following an approach applied in previous studies [7,9]. Less than 3 h of such activity per week was coded as “inadequate,” whereas at least 3 h per week was coded as “adequate.” This measure was not intended to assess the overall quality of physical activity; rather, it was treated as an operationalized indicator of intensity- and duration-based physical activity adequacy [10].

The questionnaire was pretested in a pilot study involving one general secondary school class (n = 28). The pilot study assessed the comprehensibility of the questions, the clarity of the response options, and the applicability of the instrument. The questionnaire was finalized based on the findings of the pilot study. A total of 448 students participated in the data collection. Of the returned questionnaires, 37 were excluded because they were incomplete; consequently, 411 participants were included in the final analytical sample. Missing values were not imputed. Analyses were conducted using the available data for each variable. Data were entered using Microsoft Excel 2016, and statistical analyses were performed using IBM SPSS Statistics version 30.0. Statistical significance was set at *p* < 0.05 for all analyses. Descriptive statistics were used to characterize the sample. Continuous variables are presented as the mean ± standard deviation (SD), whereas categorical variables are reported as frequencies and percentages. The internal consistency of the MSPSS subscales was assessed using Cronbach’s alpha. Associations between categorical variables were examined using the chi-square test.

Binary, ordinal, or multinomial logistic regression was applied as appropriate to the measurement level of the dependent variable. These models were used to examine associations between the structural and psychosocial characteristics of the family microenvironment and adolescents’ physical activity, BMI-derived weight status, and subjective weight perception. Family microenvironmental variables included family structure, family size, the number and sex of siblings, parental educational attainment, and perceived family support. Place of residence was included as a contextual sociodemographic factor representing the broader social and physical environment, whereas perceived peer support was examined as a complementary psychosocial factor outside the family microenvironment. Participants’ age and sex were included as covariates in all principal regression models.

Ordinal and binary logistic regression analyses were used to examine factors associated with the two physical activity indicators. A two-step hierarchical binary logistic regression analysis was conducted to examine factors associated with BMI-derived weight status. The dependent variable contrasted overweight/obesity with normal weight, with normal weight serving as the reference category; participants classified as having thinness were not included in this analysis. Model 1 included family structure, parental educational attainment, place of residence, sibling characteristics, and the number of siblings. In Model 2, the family and peer support dimensions of the MSPSS were added to the variables included in Model 1.

Multinomial logistic regression analysis was conducted to identify factors associated with subjective weight misperception, with accurate perception serving as the reference outcome category. Model 1 included family structure, parental educational attainment, place of residence, sibling characteristics, and the number of siblings. A second adjusted model included maternal educational attainment, place of residence, BMI-derived weight-status categories, and the family and peer support dimensions of the MSPSS. For BMI-derived weight status, normal weight served as the reference category.

Results are reported as adjusted odds ratios (AORs) with 95% confidence intervals (95% CIs). Overall model fit was evaluated using the likelihood-ratio test, goodness-of-fit statistics, and Nagelkerke’s pseudo-*R*^2^. For ordinal logistic regression, the proportional odds assumption was evaluated using the test of parallel lines; for binary logistic regression, model calibration was assessed using the Hosmer–Lemeshow test.

Results are reported as odds ratios (ORs) with 95% confidence intervals (95% CIs). Model fit was evaluated using the likelihood-ratio test and Nagelkerke’s R^2^.

Written permission to conduct the study was granted by the principal of the participating school and remained valid until 24 April 2025. Data collection was completed on 15 April 2025. Written information about the study was provided to the students’ parents or legal guardians, and a passive parental consent procedure was applied. Student participation was voluntary, the questionnaires were completed anonymously, and no personally identifiable information was collected.

Written information about the purpose and procedures of the study and the processing of data was provided to the parents or legal guardians of all eligible students. Passive parental consent was applied: parents or legal guardians were given the opportunity to decline their child’s participation, and students for whom a parental objection was received were not included in the study. Participation was voluntary, and the students provided assent by agreeing to complete the anonymous questionnaire. The questionnaires contained no personally identifiable information. Following data processing, the findings were reported exclusively in aggregate form; therefore, no individual student, class, or institution could be identified. Data were processed in accordance with Regulation (EU) 2016/679 of the European Parliament and of the Council, the General Data Protection Regulation.

## 3. Results

### 3.1. Sample

The sociodemographic characteristics of the adolescents participating in the study are presented in detail in Table 1. The mean age of the respondents was 16.86 years (SD = 0.82; range = 14–18 years), indicating a relatively homogeneous age distribution. Regarding place of residence, participants were relatively evenly distributed across the different settlement types, with those living in villages or other rural settlements representing the largest group.

For both parents, completion of secondary education with a school-leaving examination was the most frequently reported level of educational attainment. No parents in the sample had primary education or lower as their highest level of educational attainment. However, the proportion of participants whose mothers had a tertiary qualification was higher than the corresponding proportion for fathers. Regarding family structure, the vast majority of adolescents lived in two-parent families, and most households consisted of no more than four members. Most respondents had at least one sibling. The number of siblings ranged from 0 to 5 (M = 0.97; SD = 0.832).

The study included 411 adolescents, of whom 138 (33.4%) were boys and 273 (66.6%) were girls. Detailed sociodemographic, family-related, and physical activity characteristics of the participants, stratified by sex, are presented in Supplementary Table S2.

Perceived social support was measured using the Multidimensional Scale of Perceived Social Support (MSPSS). Descriptive analyses indicated that the mean level of perceived social support received from family was high (M = 17.54; SD = 3.33; range = 4–20). The perceived level of social support received from friends was somewhat lower and showed less variability (M = 13.54; SD = 2.21; range = 3–15).

Regarding the frequency-based physical activity indicator, 21.7% of the respondents reported at least 60 min of moderate-to-vigorous physical activity on all seven days of the previous week. A further 31.4% reported this amount of activity on 5–6 days, 31.1% on 3–4 days, and 15.8% on no more than 2 days of the previous week. The intensity- and duration-based adequacy of physical activity was operationalized as engagement in at least three hours per week of leisure-time physical activity at an intensity sufficient to induce sweating. In the present study, this measure was not interpreted as an indicator of the overall quality of physical activity but as an operationalized indicator jointly reflecting its intensity and duration. Overall, 67.2% of the sample met this criterion, whereas the remaining 32.8% were classified as having inadequate physical activity.

### 3.2. Associations of Family Microenvironmental Characteristics and Social Support with Physical Activity Frequency and Intensity- and Duration-Based Adequacy Among Adolescents

Two separate logistic regression models were constructed to examine the sociodemographic and family background factors associated with physical activity. The variables entered into the models included family type, household size, the number and sex of siblings, parental educational attainment, and place of residence. Associations between family microenvironmental factors and physical activity were examined using models adjusted for age and sex (Table 2).

None of the family microenvironmental characteristics included in the model were significantly associated with physical activity frequency (all *p* > 0.05); however, sex and age, which were included as covariates, showed statistically significant associations. The first model examined the frequency-based physical activity indicator using ordinal logistic regression. The model yielded a Nagelkerke pseudo-*R*^2^ of 0.136, and the test of parallel lines confirmed that the proportional odds assumption was satisfied (*p* = 0.921). Of the variables included in the model, only the two covariates were statistically significant. Sex showed a significant association with physical activity frequency (*B* = 1.053, 95% CI [0.546, 1.560], *p* < 0.05), indicating higher physical activity levels among boys. Age was also significantly and negatively associated with physical activity frequency (*B* = −0.477, 95% CI [−0.754, −0.200], *p* < 0.05), indicating that physical activity levels decreased with increasing age. None of the family background characteristics—family structure, family size, the sex or number of siblings, parental educational attainment, or place of residence—was significantly associated with physical activity frequency in this model (all *p* > 0.05).

The second model examined the intensity- and duration-based physical activity adequacy indicator (adequate vs. inadequate) using binary logistic regression. The model yielded a relatively low Nagelkerke pseudo-*R*^2^ of 0.066, while the Hosmer–Lemeshow test indicated an acceptable fit between the observed and model-estimated values (*p* = 0.065). Neither the sociodemographic and family background variables nor sex or age were significantly associated with intensity- and duration-based physical activity adequacy (all *p* > 0.05).

Logistic regression analyses were used to examine the extent to which the dimensions of perceived social support—family and peer support—were associated with the frequency-based and the intensity- and duration-based indicators of physical activity (Table 3).

In Model 1, which examined the frequency-based physical activity indicator in relation to perceived social support while controlling for sex and age, the proportional odds assumption was satisfied, as indicated by the test of parallel lines (*p* = 0.364). The model yielded a Nagelkerke pseudo-*R*^2^ of 0.103. Neither perceived family support nor perceived peer support were significantly associated with physical activity frequency. By contrast, sex (*B* = −1.090, *p* < 0.001) and age (*B* = −0.328, *p* < 0.001) showed significant negative associations with the outcome. Older participants and girls reported significantly lower levels of physical activity.

A binary logistic regression model was used to examine the intensity- and duration-based physical activity adequacy indicator (adequate vs. inadequate) in relation to the dimensions of perceived social support. This second model yielded a very low Nagelkerke pseudo-*R*^2^ of 0.011. Nevertheless, the Hosmer–Lemeshow test indicated an acceptable fit between the observed and model-estimated values (*p* = 0.626). Neither perceived family support nor perceived peer support were significantly associated with intensity- and duration-based physical activity adequacy. The covariates, sex and age, were also nonsignificant in this model.

Overall, neither dimension of perceived social support was significantly associated with physical activity frequency or intensity- and duration-based physical activity adequacy in the study sample. Sex and age were significantly associated only with the frequency-based physical activity indicator.

### 3.3. Associations of the Family Microenvironment and Social Support with Nutritional Status

Based on the classification of self-reported anthropometric data using sex- and age-specific BMI percentiles, the majority of the sample (57.4%) had normal nutritional status. However, the prevalence of excess weight was high: more than one-quarter of the adolescents (26.7%) were classified as having overweight or obesity. Adolescents classified as thin accounted for 15.9% of the sample. The supplementary analysis based on the 2007 WHO BMI-for-age reference yielded a similar distribution, although the prevalence estimates differed: 12.5% of participants were classified as having thinness, 64.4% as having normal weight, and 23.1% as having overweight or obesity (Supplementary Table S3). Subjective weight perception was assessed using a five-point Likert-type item. By comparing the responses with the BMI-percentile categories, participants were classified into three distinct groups. The analysis showed that only slightly more than half of the adolescents (55.2%) accurately perceived their nutritional status. Weight misperception occurred in approximately equal proportions in both directions: 22.1% underestimated and 22.7% overestimated their actual weight status.

Based on BMI-for-age percentiles, 30.9% of boys (*n* = 42) and 24.7% of girls (*n* = 67) were classified as having overweight or obesity. Regarding subjective weight perception, 37.8% of boys (*n* = 51) and 14.4% of girls (*n* = 39) underestimated their BMI-derived weight status, whereas overestimation was more prevalent among girls than among boys (30.2%, *n* = 82 vs. 7.4%, *n* = 10, respectively). Detailed descriptive data stratified by sex are presented in Supplementary Table S2. Overestimation was most prevalent among adolescents classified as thin: 62.3% perceived themselves as having a normal weight or overweight, and only 32.8% accurately perceived their weight status. Adolescents in the normal BMI-percentile category had the highest prevalence of accurate weight perception (71.2%). Nevertheless, misperception was also evident in this group: 17.7% of adolescents with a normal weight overestimated their weight, while 11.1% underestimated it.

Among adolescents with overweight or obesity, the direction of misperception was reversed. Almost half (48.1%) underestimated their nutritional status, perceiving themselves as thinner than they actually were and most commonly as having a normal weight despite their BMI-derived overweight or obesity. In this group, 44.3% accurately perceived their nutritional status, whereas overestimation was uncommon (7.5%).

A chi-square test was used to examine the association between BMI-derived weight status based on BMI-percentile categories and subjective weight perception. A significant association was found between the BMI-derived weight-status categories and subjective weight perception (χ^2^ = 134.231; *p* < 0.001), indicating that adolescents’ perceptions of their body weight may differ substantially from their actual anthropometric status. Overall, the findings demonstrate that although more than two-thirds of adolescents with a normal weight had an accurate weight perception, misperception became more prevalent toward the extremes of the nutritional-status distribution. Adolescents classified as thin tended to overestimate their weight, whereas a substantial proportion of those with overweight or obesity underestimated their actual nutritional status.

A two-step hierarchical binary logistic regression analysis was conducted to identify factors associated with BMI-derived weight status, contrasting overweight/obesity with normal weight, with the normal-weight group serving as the reference category. In Model 1, adolescents living in single-parent families had higher odds of having overweight or obesity than those living in two-parent families (AOR = 3.26; 95% CI [1.43, 7.48]; *p* < 0.01). Maternal vocational or vocational secondary educational attainment showed an inverse exploratory association with overweight/obesity compared with tertiary education (AOR = 0.31; 95% CI [0.10, 0.97]; *p* < 0.05).

After the social-support dimensions were added in Model 2, the estimated association for single-parent family structure remained similar (AOR = 3.48; 95% CI [1.51, 8.05]; *p* < 0.01). Maternal vocational or vocational secondary educational attainment remained inversely associated with overweight/obesity (AOR = 0.29; 95% CI [0.09, 0.89]; *p* < 0.05), whereas paternal vocational or vocational secondary educational attainment was associated with higher odds of overweight/obesity compared with tertiary education (AOR = 2.70; 95% CI [1.03, 7.09]; *p* < 0.05). The addition of the social-support dimensions increased Nagelkerke’s R^2^ only marginally, from 12.2% to 13.2%. However, neither model was statistically significant overall (Model 1: *p* = 0.110; Model 2: *p* = 0.161); therefore, the individual coefficients should be considered exploratory and interpreted cautiously (Table 4).

A two-step multinomial logistic regression analysis was conducted to identify factors associated with subjective weight misperception, with accurate weight perception serving as the reference outcome category. Model 1, which examined family microenvironmental factors, was not statistically significant overall (*p* = 0.254; Nagelkerke’s R^2^ = 0.120).

In the second step, BMI-derived weight-status categories were included as covariates, together with place of residence, maternal educational attainment, and the family- and peer-support dimensions of the MSPSS. Model 2 was statistically significant (*p* < 0.001) and yielded a Nagelkerke pseudo-*R*^2^ of 0.401. The goodness-of-fit tests indicated an adequate model fit (Pearson: *p* = 0.151; deviance: *p* = 0.922).

BMI-derived weight status showed the strongest association with weight misperception. Compared with adolescents classified as having normal weight, those classified as having overweight or obesity had more than ninefold higher odds of underestimating their weight status rather than perceiving it accurately (AOR = 9.33; 95% CI [5.15, 16.90]; *p* < 0.001). Conversely, adolescents classified as having thinness had more than eightfold higher odds of overestimating their weight status rather than perceiving it accurately (AOR = 8.17; 95% CI [4.19, 15.92]; *p* < 0.001).

After adjustment for BMI-derived weight status, higher perceived peer support was associated with lower odds of weight underestimation. Each one-point increase in the peer-support score was associated with 15% lower odds of underestimating rather than accurately perceiving one’s weight status (AOR = 0.85; 95% CI [0.74, 0.97]; *p* = 0.019). Perceived family support showed a nonsignificant association with lower odds of weight overestimation (AOR = 0.67; 95% CI [0.44, 1.00]; *p* = 0.051). Because this result did not reach the predefined threshold for statistical significance, it should be interpreted only as a tentative trend. Compared with adolescents living in the capital or a county seat, those living in villages had more than twice the odds of overestimating rather than accurately perceiving their BMI-derived weight status (AOR = 2.53; 95% CI [1.25, 5.12]; *p* = 0.010). The corresponding association for adolescents living in towns was not statistically significant (AOR = 1.73; 95% CI [0.82, 3.67]; *p* = 0.149) (Table 4).

## 4. Discussion

The aim of this study was to analyze adolescents’ physical activity, BMI-derived weight status, and subjective weight perception, with particular emphasis on the structural characteristics of the family microenvironment and perceived social support.

In the present sample, 21.7% of adolescents reported at least 60 min of moderate-to-vigorous physical activity on all seven days of the previous week. This proportion is similar to the 21.3% reported in the 2022 Hungarian HBSC survey using the same seven-day frequency criterion [7]. However, this indicator should not be interpreted as a direct measure of adherence to the current WHO recommendation, which specifies an average of at least 60 min per day across the week. The findings nevertheless indicate that regular physical activity was limited in a substantial proportion of the examined adolescents. Regarding the intensity- and duration-based indicator of physical activity adequacy, more than two-thirds of respondents (67.2%) engaged in at least 3 h of exercise per week at an intensity sufficient to induce sweating, which is also consistent with HBSC trends [7,48].

The international literature emphasizes the importance of parental background. Maric et al. identified parental educational attainment as a positive predictor of physical activity [20], while Bartusné Szmodis et al. reported that children of parents with tertiary education participated in sports more frequently [21]. In contrast, the sociodemographic and family background variables included in our logistic regression models explained little of the variance in physical activity. Langøy et al. and Fan et al. identified a two-parent family structure as a protective factor for meeting movement behavior recommendations [23,24]; however, this association was not significant in our sample.

Neither perceived family support nor perceived peer support were significantly associated with either physical activity indicator after adjustment for age and sex. This finding differs somewhat from the Hungarian results reported by Karácsony et al., according to which adolescents engaging in regular physical activity of adequate intensity reported significantly higher levels of family social support [32]. However, our findings support the conclusion of the systematic review by Larsen et al. that the available theoretical and empirical evidence remains insufficient to establish a direct effect of family support on children’s diet and physical activity [29]. Another Hungarian study demonstrated that the direct parental influence on sport selection and sport specialization was generally moderate or low; however, greater family financial investment in sports was associated with stronger parental influence [19]. The absence of statistically significant associations may reflect the characteristics of the sample, the operationalization of physical activity and social support, or other unmeasured contextual factors; it should therefore not be interpreted as evidence that family or peer support is unrelated to adolescent physical activity.

These findings suggest that although parental role modeling and support are important in shaping children’s lifestyles [21] and a supportive family environment contributes to children’s psychological well-being and academic achievement [31], the mechanisms through which family support influences physical activity are likely to be more complex and require further investigation.

Future intervention studies could examine whether daily physical education and recreational forms of activity help adolescents maintain adequate levels of physical activity and develop positive attitudes toward an active lifestyle. Such studies may also consider the needs of less active subgroups [5] and evaluate recreational and experience-based forms of physical activity extending beyond competitive sports, as these approaches may be more sustainable during the transition to adulthood [6,49].

Childhood and adolescent overweight and obesity are multifactorial conditions arising from interactions among biological, psychological, and environmental factors, including genetic susceptibility, unhealthy dietary habits, physical inactivity, and excessive screen use [14,15]. In our sample, the combined prevalence of overweight and obesity approached 30% (26.7%), while 15.9% of participants were classified as thin. When interpreting the prevalence of weight-status categories, it is important to recognize that Hungarian and international reference standards may produce different classification results. In the present study, participants were classified using both Hungarian age- and sex-specific BMI percentiles and the 2007 WHO BMI-for-age reference. Although the two reference standards produced broadly similar distribution patterns, the resulting prevalence estimates differed. This finding highlights the need to consider the reference standard used when making international comparisons of weight status among adolescents. The Hungarian results of the Childhood Obesity Surveillance Initiative (COSI) reported a similar prevalence of excess weight of approximately 27%, placing Hungary in the middle range among European countries [50]. Szűcs et al. also reported a comparable proportion of children affected by excess weight based on school health screening data [51].

The exploratory individual coefficients suggested possible associations between selected structural characteristics of the family microenvironment and BMI-derived weight status. Single-parent family structure and lower paternal educational attainment were associated with higher odds of overweight/obesity, whereas maternal vocational or vocational secondary educational attainment showed an inverse association. However, neither overall regression model was statistically significant. These individual coefficients should therefore be interpreted as exploratory findings that require confirmation in larger, representative, and longitudinal studies and should not be used to define priority intervention groups.

Adolescence is a critical period for establishing dietary habits that may persist into adulthood and contribute to the subsequent development of obesity. The transition from adolescence to adulthood is characterized by increasing independence, autonomy, and responsibility. It is often the first period during which individuals independently decide how, what, where, and when to eat; therefore, it is crucial for establishing lifelong healthy dietary patterns [52]. Future studies could evaluate whether after-school or community-based recreational programs and cooking clubs may help address socioeconomic inequalities and support autonomous, healthy decision-making among adolescents.

One of the most important findings of our study was the discrepancy between BMI-derived weight status and subjective weight perception. Only 55.2% of adolescents accurately perceived their nutritional status. Danford et al. emphasized that weight perception not only affects the successful maintenance of an optimal body weight but also fundamentally determines whether young people perceive lifestyle changes as necessary [33]. When weight perception is inaccurate, motivation to modify lifestyle behaviors may be reduced.

In our sample, the chi-square test demonstrated a significant association between the objective and subjective categories, with substantial weight misperception among adolescents with weight-related problems. Among adolescents classified as thin, 62.3% overestimated their weight, whereas almost half of those with overweight or obesity (48.1%) underestimated their actual weight status. This underestimation is consistent with the US findings of Sarafrazi et al., according to which 77% of children with overweight and 43% of those with obesity perceived themselves as having a healthy weight [36]. It is also consistent with the European findings of Yang et al., who reported that a substantial proportion of objectively overweight adolescents did not perceive themselves as overweight [37].

In a study involving more than 87,000 participants, Park similarly found that more than one-quarter of adolescents inaccurately perceived their weight status and identified substantial sex differences: underestimation was more common among boys, whereas overestimation was more common among girls [38]. This pattern was also confirmed by López-Bueno et al. [39]. These findings suggest that future school-based health-promotion research may examine approaches addressing misconceptions about obesity and inaccurate weight perception among adolescents [39]. Such research could also evaluate the provision of information about objective criteria for a healthy body weight [40].

Conversely, weight overestimation is a risk factor for unhealthy weight-control behaviors. Future studies may benefit from assessing adolescents’ perceived weight and body-image accuracy alongside directly measured anthropometric indicators to obtain a more comprehensive understanding of weight-related health during adolescence.

Exploratory individual coefficients suggested possible associations between selected family characteristics and BMI-derived weight status, whereas perceived peer support was associated with lower odds of weight underestimation in the adjusted weight-perception model. Perceived family support showed a nonsignificant tendency toward lower odds of weight overestimation; therefore, this finding should be interpreted cautiously and requires confirmation in further studies. This pattern is broadly consistent with Ben-Yaish et al., who found that overestimation was associated with lower family support and poorer parent–child communication [40]. Supportive family relationships, positive parent–child communication, and emotional security have been shown to positively influence adolescents’ health perceptions and self-esteem [41,42]. Moreover, greater support from parents and teachers has been associated with more accurate weight perception [40].

Future intervention studies could examine whether strengthening supportive communication among parents, teachers, and adolescents contributes to more accurate weight perception. Such studies could evaluate communication-focused components delivered through parent meetings or professional-development programs for teachers.

Higher perceived peer support was associated with lower odds of weight underestimation after adjustment for BMI-derived weight status. Each one-point increase in the peer-support score was associated with 15% lower odds of underestimating rather than accurately perceiving one’s weight status. Although this association was identified in a statistically significant overall model, the cross-sectional design does not allow causal or protective effects to be inferred. The finding therefore warrants confirmation before implications for peer-based interventions can be established. Alternative explanations, including reverse-direction associations and residual confounding by unmeasured individual or social factors, cannot be excluded.

Overall, our findings indicate that adolescent health behavior is not determined exclusively by either family structure or the presence of social support. Instead, these factors appear to have different associations with physical activity, nutritional status, and weight perception. Exploratory individual coefficients suggested possible associations between selected family characteristics and BMI-derived weight status, whereas perceived peer support was associated with lower odds of weight underestimation in the adjusted weight-perception model. These findings support further research into integrated approaches addressing physical activity, accurate weight perception, and supportive family and peer environments.

## 5. Limitations

The findings of this study should be interpreted in light of several limitations. First, the cross-sectional design does not permit causal conclusions and allows only the identification of associations. Second, height and body weight were self-reported, although participants were asked to provide the values communicated to them following their most recent school health screening. These data were neither obtained directly from the healthcare professionals who performed the measurements nor verified against official school health records. Consequently, recall and reporting bias, including the possible overestimation of height and underestimation of body weight, cannot be excluded and may have affected BMI calculation and weight-status classification. Third, although BMI is widely used in epidemiological research, it does not provide direct information about body composition, body-fat distribution, or central adiposity. The primary use of Hungarian age- and sex-specific BMI reference values may also limit the direct international comparability of the prevalence estimates, although a supplementary classification based on the 2007 WHO BMI-for-age reference was provided.

Furthermore, convenience sampling and the restriction of data collection to secondary school students in Western Transdanubia limit the generalizability of the findings. Possible participant overlap with an earlier survey conducted in some of the same schools cannot be excluded or quantified because both data collections were anonymous and no persistent participant identifiers were used. Consequently, the present sample cannot be confirmed as completely independent at the individual level. Finally, several regression models had limited explanatory performance or nonsignificant overall model tests. Therefore, statistically significant individual coefficients identified in these models should be considered exploratory and interpreted cautiously. Overall, the findings provide hypothesis-generating evidence that requires confirmation in larger, representative, and longitudinal studies using directly measured anthropometric data.

## 6. Conclusions

This study highlights the interconnected roles of physical activity, nutritional status, weight perception, and the family microenvironment in adolescent health. Only 21.7% of the participants reported at least 60 min of moderate-to-vigorous physical activity on all seven days of the previous week, while 26.7% were classified as having overweight or obesity. In addition, nearly half of the adolescents inaccurately perceived their weight status, with underestimation being particularly common among those with overweight or obesity and overestimation among those classified as thin.

The family microenvironment and perceived social support showed different patterns of association with the examined outcomes. Neither perceived family support nor perceived peer support were significantly associated with physical activity frequency or intensity- and duration-based physical activity adequacy. Exploratory individual coefficients suggested possible associations between selected family characteristics and BMI-derived weight status; however, the corresponding overall regression models were not statistically significant. In the adjusted weight-perception model, higher perceived peer support was associated with lower odds of weight underestimation, whereas the association between perceived family support and weight overestimation did not reach statistical significance.

These findings may inform future school- and family-based health-promotion research, but they do not provide sufficient evidence for specific intervention recommendations. Future studies could examine integrated approaches involving adolescents, parents, teachers, and peers while addressing physical activity, weight-related health knowledge, and accurate weight perception. Given the cross-sectional design, self-reported anthropometric data, convenience sampling, and the limited explanatory performance of several models, the observed associations should not be interpreted as causal. Larger, representative, longitudinal studies using directly measured anthropometric data are needed to confirm the findings. Future research should also incorporate waist circumference or other measures of central adiposity and body composition alongside BMI. 

## Figures and Tables

**Table 1:** Sociodemographic characteristics of the sample (N = 411).

Variable	Category	%	n
Sex	Male	33.4	138
	Female	66.6	273
Place of residence	County seat	31.4	128
	Town	28.6	117
	Village/rural settlement	40.0	163
Mother’s educational attainment	Vocational/vocational secondary education	13.7	55
	Secondary education with a school-leaving examination	45.0	181
	Tertiary education	41.3	166
Father’s educational attainment	Vocational/vocational secondary education	25.6	101
	Secondary education with a school-leaving examination	43.5	172
	Tertiary education	30.9	122
	Missing/“I do not know”	3.9	16
Family structure	Two-parent family	70.6	290
	Single-parent family	18.5	76
	Reconstituted family	10.9	45
Siblings	None	29.2	120
	Only brothers	31.6	130
	Only sisters	27.5	113
	Both brothers and sisters	11.7	48
Household size	1–4 persons	91.7	377
	5 or more persons	8.3	34

**Table 2:** Logistic regression models predicting the physical activity (N = 411).

Predictor	Model 1: Quantitative Indicator of Physical Activity		Model 2: Intensity- and Duration-Based Indicator of Physical Activity Adequacy	
	β	95% CI (LL, UL)	OR (Exp B)	95% CI (LL, UL)
Type of family				
Single-parent family (1)	−0.235	(−0.891, 0.421)	1.428	(0.489, 4.164)
Reconstituted family (2)	0.253	(−0.471, 0.977)	1.028	(0.476, 2.218)
Two-parent family (3) ^a^	0.00	—	1.00	—
Size of family				
Large family (1)	0.699	(−0.056, 1.453)	1.652	(0.653, 4.180)
Small family (2) ^a^	0.00	—	1.00	—
Sex of siblings				
Only brothers (1)	0.632	(−0.278, 1.542)	0.671	(0.354, 1.237)
Only sisters (2)	0.681	(−0.215, 1.576)	0.792	(0.276, 2.277)
Both brothers and sisters (3)	0.498	(0.302, 1.298)	1.645	(0.739, 3.861)
No siblings (4) ^a^	0.00	—	1.00	—
Number of siblings	0.217	(−0.468, 0.901)	1.038	(0.412, 2.615)
Mother’s educational attainment				
Vocational/vocational secondary education (1)	−0.104	(−0.958, 0.750)	0.960	(0.334, 2.686)
Upper secondary education with a school-leaving examination (2)	−0.297	(−0.824, 0.229)	0.950	(0.334, 2.699)
Tertiary education (3) ^a^	0.00	—	1.00	—
Father’s educational attainment				
Vocational/vocational secondary education (1)	0.141	(−0.592, 0.874)	0.627	(0.292, 1.345)
Upper secondary education with a school-leaving examination (2)	0.180	(−0.388, 0.747)	0.867	(0.489, 2.110)
Tertiary education (3) ^a^	0.00	—	1.00	—
Place of residence				
Village/rural settlement (1)	0.099	(−0.468, 0.666)	0.696	(0.362, 1.337)
Town (2)	0.400	(−0.181, 0.981)	0.739	(0.375, 1.457)
County seat/capital (3) ^a^	0.00	—	1.00	—
Sex	1.053 *	(0.546, 1.560)	0.756	(0.420, 1.361)
Age	−0.477 *	(−0.754, −0.200)	0.729	(0.520, 1.024)

**Note** * *p* < 0.001. LL = lower limit; UL = upper limit; CI = confidence interval; OR = odds ratio. ^a^ Reference category. 1. model Nagelkerke R^2^ = 0.136, Parallel Lines *p* = 0.921. 2. model Nagelkerke R^2^ = 0.066, Hosmer–Lemeshow *p* = 0.065.

**Table 3:** Logistic regression models predicting physical activity based on the dimensions of perceived social support (N = 411).

Predictor	Model 1: Quantitative Indicator of Physical Activity		Model 2:Intensity- and Duration-Based Indicator of Physical Activity Adequacy	
	β	95% CI (LL, UL)	OR (Exp B)	95% CI (LL, UL)
Social support scales				
Family support	0.030	(−0.031, 0.090)	0.971	(0.906, 1.041)
Peer support	0.027	(−0.064, 0.117)	0.997	(0.897, 1.108)
Sex	−1.090 *	(−1.476, −0.704)	1.097	(0.708, 1.699)
Age	−0.328 *	(−0.543, −0.113)	0.829	(0.642, 1.071)

Note * *p* < 0.001. LL = lower limit; UL = upper limit; CI = confidence interval; OR = odds ratio. 1. model Nagelkerke R^2^ = 0.103, Parallel Lines *p* = 0.364. 2. model Nagelkerke R^2^ = 0.011, Hosmer–Lemeshow *p* = 0.626.

**Table 4:** Hierarchical Binary and Multinomial Logistic Regression Models Examining Factors Associated with BMI-Derived Weight Status and Subjective Weight Perception (*N* = 411).

Predictors	Model 1: Family Microenvironmental Factors	Model 2: Psychosocial and Microenvironmental Factors	Model 1: Family Microenvironmental Factors		Model 2: Psychosocial and Microenvironmental Factors	
	Objective BMI category: Overweight/obesity vs. normal ^a^		Subjective weight perception: Underestimation vs. accurate perception ^b^	Subjective weight perception: Overestimation vs. accurate perception ^b^	Subjective weight perception: Underestimation vs. accurate perception ^b^	Subjective weight perception: Overestimation vs. accurate perception ^b^
	AOR (95% CI)	AOR (95% CI)	AOR (95% CI)	AOR (95% CI)	AOR (95% CI)	AOR (95% CI)
Family microenvironmental factors						
Family type (single-parent family) ^c^	3.26 (1.43–7.48) **	3.48 (1.51–8.05) **	1.00 (0.51–1.96)	1.18 (0.59–2.35)	—^i^	—^i^
Family type (reconstituted family) ^c^	0.60 (0.21–1.74)	0.61 (0.22–1.78)	1.42 (0.55–3.68)	0.91 (0.32–2.59)	—^i^	—^i^
Mother’s educational attainment (vocational/vocational secondary education) ^d^	0.31 (0.10–0.97) *	0.29 (0.09–0.89) *	1.10 (0.51–2.38)	0.82 (0.37–1.82)	0.78 (0.32–1.89)	0.80 (0.32–2.00)
Mother’s educational attainment (secondary education with a school-leaving examination) ^d^	0.54 (0.33–0.86)	0.24 (0.32–0.66)	0.95 (0.46–1.97)	0.78 (0.37–1.63)	0.61 (0.32–1.15)	1.18 (0.65–2.15)
Father’s educational attainment (vocational/vocational secondary education) ^e^	2.48 (0.96–6.41) *p* = 0.061	2.70 (1,03–7.09) *	0,72 (0.34–1.55)	1,01 (0.47–2.18)	—^i^	—^i^
Father’s educational attainment (secondary education with a school-leaving examination) ^e^	1.44 (0.68–3.04)	0.28 (0.71–1.50)	0.94 (0.47–1.89)	0.85 (0.41–1.74)	—^i^	—^i^
Place of residence (village/rural settlement) ^f^	1.07 (0.52–2.16)	0.82 (0.52–1.07)	0.87 (0.68–1.44)	1.22 (0.57–2.60)	1.02 (0.51–2.06)	2.53 (1.25–5.12) *
Place of residence (town) ^f^	0.82 (0.37–1.81)	0.66 (0.37–0.83)	1.49 (0.82–2.92)	1.86 (0.83–4.15)	0.96 (0.45–2.04)	1.73 (0.82–3.67)
Sex of siblings (only brothers) ^g^	0.68 (0.21–2.21)	064 (0.23–0.75)	0.88 (0.41–1.89)	0.91 (0.43–1.94)	—^i^	—^i^
Sex of siblings (only sisters) ^g^	1.19 (0.37–3.77)	0.61 (0.41–1.34)	1.02 (0.49–2.11)	1.15 (0.55–2.41)	—^i^	—^i^
Number of siblings (one or more siblings) ^h^	0.99 (0.39–2.50)	0.89 (0.35–2.3)	1.12 (0.53–2.36)	0.88 (0.42–1.84)	—^i^	—^i^
BMI category Thin status (1.00) ^a^	—^i^	—^i^	—^i^	—^i^	0.59 (0.13–2.76)	8.17 (4.19–15.92) **
BMI category Overweight/obesity status (2.00) ^a^	—^i^	—^i^	—^i^	—^i^	9.33 (5.15–16.90) **	0.49 (0.19–1.24)
Social support						
MSPSS family support		0.91 (0.87–0.99)			1.23 (0.78–1.93)	0.67 (0.44–1.00)
MSPSS peer support	—	1.06 (0.81–1.39)	—	—	0.85 (0.74–0.97) *	1.04 (0.90–1.19)
Model fit statistics						
Overall model fit (*p*-value)	*p* = 0.110	*p* = 0.161	*p* = 0.254		*p* < 0.001	
Goodness of fit (Pearson/deviance *p*-values)	0.068/0.183	0.083/0.547	0.127/0.192		0.151/0.922	
Nagelkerke’s R^2^	12.2%	13.2%	12.0%		40.1%	

**Notes:** * *p* < 0.05; ** ***p*** < 0.01. **AOR** = adjusted odds ratio; **CI** = confidence interval. ^a^ Reference category: normal BMI (0). ^b^ Reference category: accurate weight perception (0). ^c^ Reference category: two-parent family. ^d^ Reference category: tertiary education (mother). ^e^ Reference category: tertiary education (father). (In Model 1, the vocational education category had a *p*-value of 0.061, indicating an association that approached statistical significance.). ^f^ Reference category: capital/county seat. ^g^ Reference category: siblings of both sexes. ^h^ Reference category: no siblings (0). ^i^ Variables excluded from the reduced parsimonious models using backward elimination to optimize model fit.

## Data Availability

The data supporting the findings of this study are available from the corresponding author upon reasonable request. The data are not publicly available due to ethical and privacy considerations related to the participation of minors.

## Supplementary Materials

The following supporting information can be downloaded at: https://www.mdpi.com/article/10.3390/children13101315/s1, Table S1. Questionnaire Items Used in the Study and Operationalization of the Analytical Variables. Table S2. Sociodemographic, family-related, physical activity, nutritional status, and subjective weight perception characteristics of the study population by sex. Table S3. Comparison of BMI-Derived Weight-Status Categories Based on the Hungarian Reference Percentiles and the 2007 WHO BMI-for-Age Reference.

## Abbreviations

The following abbreviations are used in this manuscript:

AOR	Adjusted odds ratio
BMI	Body mass index
CI	Confidence interval
COSI	Childhood Obesity Surveillance Initiative
GDPR	General Data Protection Regulation
HBSC	Health Behaviour in School-aged Children
LL	Lower limit
MSPSS	Multidimensional Scale of Perceived Social Support
OR	Odds ratio
SD	Standard deviation
UL	Upper limit
WHO	World Health Organization
