# Supplementary material for: The Role of the Family Microenvironment in Adolescents’ Physical Activity and Nutritional Status
Source: Children (Basel). 2026 Sep 27;13(10):1315. doi: 10.3390/children13101315 (PMC13703084; doi:10.3390/children13101315)
Supplement: Supplementary file 1 [file children-13-01315-s001.zip › children-4547595-supplementary.pdf]

Supplementary Table S1. Questionnaire Items Used in the Study and Operationalization of the Analytical Variables

Variable	Question	Exact wording of the item	Original response options	Operationalization for analysis
Physical activity frequency	Q7	Think about the past 7 days and try to count how many days you were physically active for at least 60 minutes. Include physical activity performed during school physical education classes. For example, if you were active for 30 minutes in the morning and another 30 minutes in the afternoon on the same day, this counts as 60 minutes of physical activity on that day.	0–7 days	7 days; 5–6 days; 3–4 days; 0–2 days
Leisure-time physical activity	Q8	Outside school lessons, how many hours per week do you engage in leisure-time physical activity at an intensity sufficient to make you sweat?	None; approximately ½ hour; approximately 1 hour; approximately 2–3 hours; approximately 4–6 hours; ≥7 hours	Inadequate: <3 hours/week; adequate: ≥3 hours/week of physical activity at an intensity sufficient to induce sweating
Subjective weight perception	Q11	How do you perceive your body weight?	Much too thin; a little too thin; about the right size; a little too fat; much too fat	Compared with BMI-derived weight status: accurate perception; underestimation; overestimation
Perceived family support	Q12/3, 4, 6, 9	3. I can always count on my family.<br>4. I receive the emotional help and support I need from my family.<br>6. I can talk about my problems with my family.<br>9. My family is willing to help me make decisions.	Five-point Likert scale	Mean item score
Perceived peer support	Q12/5, 7, 10	5. I can always count on my friends.<br>7. I have friends with whom I can share my joys and sorrows.	Five-point Likert scale	Mean item score

Variable	Question	Exact wording of the item	Original response options	Operationalization for analysis
		10. I can talk about my problems with my friends.		
Mother's educational attainment	Q5	What is your mother's highest level of educational attainment?	Nine response options	Vocational/vocational secondary education; upper secondary education with a school-leaving examination; tertiary education
Father's educational attainment	Q5	What is your father's highest level of educational attainment?	Nine response options	Vocational/vocational secondary education; upper secondary education with a school-leaving examination; tertiary education

Supplementary Table S2. Sociodemographic, family-related, physical activity, nutritional status, and subjective weight perception characteristics of the study population by sex

Variable		Male		Female	
		%	n	%	n
**Place of residence**	County seat	35.8	48	28.9	80
	Town	29.1	39	28.6	78
	Village/rural settlement	35.1	47	42.5	116
**Mother's educational attainment**	Vocational/vocational secondary education	6.8	9	17.2	46
	Secondary education with a school-leaving examination	42.1	56	46.6	125
	Tertiary education	51.1	68	36.2	98
**Father's educational attainment**	Vocational/vocational secondary education	15.6	20	30.5	81
	Secondary education with a school-leaving examination	44.5	57	42.9	115
	Tertiary education	39.8	51	26.7	71
**Family structure**	Two-parent family	78.1	107	66.7	182
	Single-parent family	15.3	21	20.1	55
	Reconstituted family	6.6	9	13.2	36

Variable		Male		Female	
		%	n	%	n
**Siblings**	None	30.7	42	28.5	78
	Only brothers	33.6	46	30.8	84
	Only sisters	26.2	36	28.2	77
	Both brothers and sisters	9.5	13	12.5	35
**Household size**	1–4 persons	92.7	127	91.2	250
	5 or more persons	7.3	10	8.8	24
**Frequency-based physical activity indicator**	7 days/week	33.6	46	15.8	43
	5–6 days/week	36.4	50	28.9	79
	3–4 days/week	23.4	32	34.8	95
	0–2 days/week	6.6	9	20.5	56
**Intensity- and duration-based indicator of physical activity adequacy**	Adequate	65.7	90	67.8	185
	Inadequate	34.3	47	32.2	89
**BMI percentiles**	Underweight	11.0	15	18.1	50
	Normal	58.1	79	57.2	155
	Overweight or obesity	30.9	42	24.7	67
**subjective weight perception**	Underestimate actual weight status	37.8	51	14.4	39
	Accurately perceive nutritional status	54.8	74	55.4	150
	Overestimate actual weight status	7.4	10	30.2	82

Note: Percentages are calculated within sex. Due to missing data, the number of participants may vary across variables.

Supplementary Table S3. Comparison of BMI-Derived Weight-Status Categories Based on the Hungarian Reference Percentiles and the 2007 WHO BMI-for-Age Reference

**Weight-status category**	**Hungarian BMI percentiles, *n* (%)**	**2007 WHO BMI-for-age reference, *n* (%)**
Thinness	65 (15.9%)	51 (12.5%)
Normal weight	234 (57.4%)	263 (64.4%)
Overweight/obesity	109 (26.7%)	94 (23.1%)

**Weight-status category**	**Hungarian BMI percentiles, *n* (%)**	**2007 WHO BMI-for-age reference, *n* (%)**
Total with evaluable BMI data	408 (100%)	408 (100%)
Missing BMI data	3	3
Total sample	411	411

Note: Weight-status classification was possible for 408 participants. Evaluable BMI data were unavailable for three participants. Classification according to the 2007 WHO BMI-for-age reference [4] was performed as a supplementary analysis to improve international comparability.
